# Anxiety and depression in athletes assessed using the 12-item General Health Questionnaire (GHQ-12) - a systematic scoping review

**DOI:** 10.17159/2078-516X/2021/v33i1a10679

**Published:** 2021-01-15

**Authors:** N Armino, V Gouttebarge, S Mellalieu, R Schlebusch, JP van Wyk, S Hendricks

**Affiliations:** 1 Division of Exercise Science and Sports Medicine, Department of Human Biology, Faculty of Health Sciences, University of Cape Town, Cape Town, South Africa; 2 Health, Physical Activity, Lifestyle and Sport (HPALS) Research Centre, University of Cape Town, Cape Town, South Africa; 3 Amsterdam UMC, University of Amsterdam, Department of Orthopaedic Surgery, Amsterdam Movement Sciences, Meibergdreef 9, Amsterdam, Netherlands; 4 Amsterdam Collaboration on Health & Safety in Sports (ACHSS), Amsterdam UMC IOC Research Center of Excellence, Amsterdam, Netherlands; 5 Cardiff School of Sport and Heath Sciences, Cardiff Metropolitan University, Cardiff, United Kingdom; 6 South African Cricketers’ Association, Cape Town, South Africa; 7 Institute for Sport, Physical Activity and Leisure, Leeds Beckett University, Leeds, United Kingdom

**Keywords:** mental health, elite athletes, sport, well-being

## Abstract

**Background:**

The poor mental health of athletes is a major concern in sport. Typically, the incidence/prevalence of mental health symptoms in athletes is studied using symptom-specific questionnaires. For symptoms of depression/anxiety, one such self-reporting questionnaire is the 12-item General Health Questionnaire (GHQ-12).

**Objectives:**

The aim of this review was to synthesise and compare studies using the GHQ-12 in athletes to inform future research bodies by identifying trends and gaps in the literature.

**Methods:**

A systematic search of five electronic databases (Google Scholar, PubMed, PsychINFO, Scopus and Web of Science) was conducted on all published studies up to 1 January 2019. Inclusion criteria: (1) participants were able-bodied athletes; (2) studies measured anxiety/depression using the GHQ-12; (3) studies were full original articles from peer-reviewed journals; (4) studies were published in English.

**Results:**

Thirty-two studies were included in the review. The prevalence and incidence of symptoms of anxiety/depression ranged from 21–48% and 17–57%, respectively. The majority of studies screening anxiety/depression using the GHQ-12 were cross-sectional. Almost 70% of the studies used the traditional scoring method. The majority of study populations sampled all-male cohorts comprising football (soccer) players.

**Conclusion:**

The traditional scoring of 0-0-1-1 should be used with the cut-off set at ≥3. Also, the mean GHQ-12 score should be reported. Potential risk factors for symptoms of anxiety/depression (i.e. recent adverse life events, injury and illness, social support, pressure to perform and career transitioning) and a lack of prospective studies were identified. Future research should also broaden the spectrum of athlete populations used and aim to improve response rates.

Poor mental health of current and retired athletes is a major concern in sport.^[1–7]^ Typically, the incidence/prevalence of mental health symptoms in athletes is studied using symptom-specific questionnaires. For symptoms of anxiety and depression (often comorbid), one such self-reporting questionnaire is the 12-item General Health Questionnaire (GHQ-12). The GHQ-12 is a popular self-reporting measure of mental health.^[8]^ The GHQ has been used in community, clinical and sport settings.^[9,10]^ The original GHQ consists of 60 items, while the shorter more popular version contains only 12 items (GHQ-12).^[11–13]^ The popularity of the GHQ-12 can be attributed to its robust psychometric properties and being quick and unobtrusive to administer.^[11–13]^ The GHQ-12 includes six positively phrased items (e.g. ‘Have you been able to concentrate on what you were doing’) and six negatively worded items (e.g. ‘Have you lost much sleep over worry’). The GHQ-12 can be scored in several ways. For the traditional GHQ scoring method (0-0-1-1), items are answered on a 4-point scale with the response categories and scoring values for the positive items representing: ‘not at all’=1, ‘no more than usual’=1, ‘rather more than usual’=0, and ‘much more than usual’=0; and those for the negative items ‘not at all’=0, ‘no more than usual’=0, ‘rather more than usual’=1, and ‘much more than usual’=1.^[14]^ The scores are summed to obtain a total score between 0–12. A score of ≥2 indicates symptoms of anxiety/depression.^[9,11]^ Goldberg also suggested that to obtain an optimal trade-off between sensitivity and specificity, the mean score of the group could be used as a threshold.^[15]^ The less frequently used Likert-type scale scoring (0-1-2-3) can also be used. The scores are summed to obtain a total score between 0–36, with higher scores indicative of lower psychological well-being. Because of the larger scoring range compared to the traditional scoring method, the Likert-type scale scoring is potentially more sensitive in detecting changes in psychological well-being over time.^[11]^

While a number of reviews on the mental health of elite athletes are available in the literature, ^[2–5]^ no review currently exists which focuses specifically on anxiety and depression using the GHQ-12. One narrative review provided an overview of the prevalence and risk factors for depression but failed to discuss the tools used to measure depression.^[5]^ Other reviews have a broad scope, covering many mental health symptoms and psychological well-being behaviours (e.g. sleep disorders, ADHD/ADD, eating disorders).^[2–7]^ This makes it difficult to compare studies to develop interventions for depression and anxiety. In response, a scoping review of the literature focusing on studies using the GHQ-12 was performed. The aim of this review was to synthesise and compare studies using the GHQ-12 in athletes in order to inform future research by identifying trends and gaps in the literature.

## Methods

### Search

**Fig. 1:**
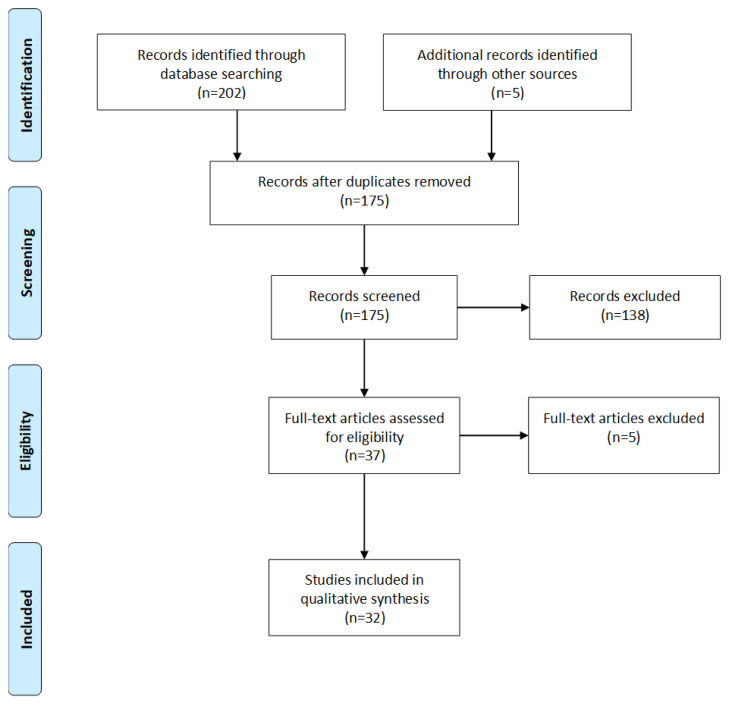
Schematic of the literature search according to PRISMA guidelines

This review was reported in accordance with the Preferred Reporting Items for Systematic Reviews and Meta-Analyses (PRISMA) guidelines. A systematic search of five electronic databases (Google Scholar, PubMed, PsychINFO, Scopus and Web of Science) was conducted on all published studies up to 1 January 2019. Key terms included: anxiety, depression, mental health, elite athletes, athletes, sport, general health questionnaires, GHQ and combinations thereof. Inclusion criteria for the review were: (1) participants were able-bodied athletes; (2) studies measured anxiety/depression using the GHQ-12; (3) studies were full, original articles from peer-reviewed journals; (4) studies were published in English. Studies were excluded if athlete and non-athlete populations were combined as a single group. Athletes are defined in this review as individuals who train in sports aiming to improve their performance; are actively participating in sport competitions; registered in a local, regional or national sports federation; and devote several hours on most days to their sport. Studies were screened at the title and abstract level for eligibility. If a decision on the eligibility of the study was unclear at the title and abstract level, the full text was retrieved and screened. After merging the databases and removing duplicates, a second author screened the studies for reliability purposes. A schematic of the process is shown in Fig. 1.

### Data extraction

The authors, year of publication, study design, purpose of the study, study population characteristics (sample size, age, active/retired, ratio of men to women, country), GHQ-12 (scoring method, cut-off point, mean score), prevalence and incidence of anxiety/depression, as well as factors associated with anxiety/depression were extracted from each study and tabulated.

## Results

A total of 202 studies were identified through database searching, while five were retrieved through other sources. After duplicates were removed, 175 studies were screened by title/abstract, of which 37 full texts were assessed. Finally, 32 studies were included in the review (Table 1).

### Study design

The majority of studies used either a cross-sectional (56%, n=18),^[9,16–32]^ or a prospective design (38%, n=12).^[33–44]^ One study used a randomised controlled trial design^[45]^ and another used a quasi-experimental design.^[46]^

### Population characteristics

The majority of studies were conducted on football players (50%, n=16). Other sports that have used the GHQ-12 are rugby union (16%, n=5), cricket (9%, n=3), ice hockey (9%, n=3), and Gaelic football (6%, n=2). Only one study has been published on Australian football players, distance runners, handball players, and horse jockeys, respectively. The majority of studies included all-male cohorts (69%, n=22), while only one study included an all-female cohort. Several studies included mixed cohorts (22%, n=7), while a couple did not specify the sex of the participants (6%, n=2). The majority of studies included adult athletes (81%, n=26), while some used only adolescent athletes (13%, n=4). A few studies included both adult and adolescent athletes (6%, n=2). Most of the studies consisted of active athletes (63%, n=20). Several studies included retired athletes exclusively (19%, n=6), while others included both active and retired athletes (19%, n=6). Most of the included studies used elite athletes, where this was defined as professional, international/national or Olympic-level athletes (84%, n=27). Non-elite athletes in other studies included university, adolescent/high school, or local/regional athletes (16%, n=5).

### Prevalence/incidence

The prevalence of symptoms of anxiety/depression assessed by the GHQ-12 ranged from 21–48%. The incidence of symptoms of anxiety/depression ranged from 17–57%.

### GHQ scoring method and cut-off points

The studies (72%, n=23) used mainly the ‘traditional’ GHQ scoring method (0-0-1-1), while 19% (n=6) used the Likert-scale scoring method (0-1-2-3). The remaining studies (9%, n=3) did not specify the GHQ scoring method. Of the 23 studies that used the traditional GHQ scoring method, 43% (n=10) set the cut-off at ≥two, another 43% (n=10) at ≥3 and two studies (9%) set the cut-off at ≥4. One study did not specify the cut-off point for indicating symptoms of anxiety/depression.

## Discussion

The GHQ-12 is a popular tool used to screen the presence of anxiety/depression symptoms among athletes. Its popularity can be attributed to its robust psychometric properties and quick unobtrusive administration.^[11–13]^ This is the first review to focus specifically on the GHQ-12 in order to compare studies and identify potential risk factors for anxiety/depression, as well as methodological considerations for future research. Not surprisingly, methodological inconsistencies between studies using the GHQ-12 were found. Sixty-eight percent (n=23) of the studies used the traditional 0-0-1-1 scoring method. Of these 23 studies, three different cut-offs were applied. For future research, we recommend the traditional scoring of 0-0-1-1 be used. Furthermore, to improve anxiety/depression prevalence and incidence comparisons, we also suggest that the recommended cut-off for the GHQ-12 for athletes be set at ≥3. In addition, the mean GHQ-12 score should be reported, as suggested by Goldberg.^[15]^

Most of the studies used a cross-sectional or prospective cohort design with the objective of determining the prevalence and incidence of symptoms of anxiety/depression. The prevalence and incidence ranged from 21–48% and 17–57%, respectively.^[9,16,17,19,23,25–34,36–40,42,44,46]^ A sample of elite athletes from the United Kingdom (cricket, fencing, hockey, rugby union and many others) and elite Gaelic athletes presented with the highest prevalence of anxiety/depression symptoms (48%),^[9,40]^ while the highest incidence was found in a sample of elite Dutch athletes (57%).^[38]^ The response rates for most of these epidemiological studies were around 30%, with 40% comprising samples of European professional football players,^[16,19,26,27,29–31,33,34,36,41,42]^ thus decreasing the generalisability of the findings and highlighting a clear area for further investigation.

Several studies have identified potential risk factors for anxiety/depression symptoms. The most notable of these are recent adverse life events,^[17,19,27,31,44]^ career dissatisfaction,^[9,26,44]^ injuries,^[19,40]^ surgeries,^[19]^ social support,^[21,31]^ osteoarthritis, pressure to perform, and career transitioning.^[21,23]^ In one study, the GHQ score was used as a predictor for musculoskeletal injury.^[42]^ Although no reported association was found between anxiety/depression symptoms and severe musculoskeletal injuries, the study was novel in using the GHQ score as a potential risk factor for predicting injury. Using the GHQ score as a predictor rather than an outcome suggests that the GHQ-12 could potentially be used as a monitoring tool for injury risk. Two studies attempted to reduce the GHQ-12 scores of athletes. Wilson et al. attempted to reduce GHQ-12 scores in 10 experienced jockeys using an exercise and diet intervention.^[46]^ However, the study was limited by its small sample size and lacked a control group. The other study used a randomised control study design, where the intervention was an internet-based cognitive behavioural therapy.^[45]^ No significant differences were found between the intervention and control group, and this was attributed to the short period of the intervention.

The GHQ-12 is proposed to measure symptoms of anxiety and depression (reported also as anxiety/depression).^[2]^ Although often comorbid, these are two different psychological conditions. Depression is a medical condition that negatively impacts on how an individual feels, thinks and acts.^[47]^ Symptoms include sadness, apathy, guilt, low self-esteem, trouble sleeping, decreased appetite, tiredness, poor concentration and suicidal ideation.^[47]^ Depression can be chronic or recurrent, and can significantly affect an individual’s ability to cope with daily life.^[48]^ Anxiety is defined as the anticipation of a future concern, whilst fear is an emotional response to an immediate threat.^[47]^ Anxiety disorders are characterised by excessive feelings of anxiety and fear.^[48]^ Researchers and sport practitioners should be aware of the distinctions between depression and anxiety when using the GHQ-12.

## Conclusion

This review compared GHQ-12 studies and identified potential risk factors for depression and anxiety, as well as methodological considerations for future research. Based on this review, we recommend the traditional scoring of 0-0-1-1 be used with the cut-off set at ≥3. Also, the mean GHQ-12 score should be reported. The prevalence and incidence of symptoms of anxiety/depression ranged from 21–48% and 17–57%, respectively. Potential risk factors for anxiety/depression include recent adverse life events,^[17,19,27,31,44]^ career dissatisfaction,^[9,26,44]^ injuries,^[19,40]^ surgeries,^[19]^ social support,^[21,31]^ osteoarthritis, pressure to perform, and career transitioning.^[21,23]^ Future research should broaden the spectrum of athlete populations and aim to improve response rates. Finally, researchers and sport practitioners should acknowledge that the GHQ-12 does not differentiate between symptoms of anxiety and depression.

## Figures and Tables

**Table 1:** Summary of General Health Questionnaire (GHQ) studies on athletes

Author	Study design	Purpose	Population; Country	N; Men/ Women %	GHQ scoring method	GHQ cut off point	Prevalence	Incidence	Predictor	Outcome	GHQ mean score	Findings
**Blakelock et al., 2016** ^[33]^	Observational, prospective cohort	Psychological distress following deselection in soccer players	Elite adolescent footballers, 15–18 years (16.31 ± 1.10); UK	91; 100/0	GHQ method	3	36% (time point 1; n=14)	55% (time point 3; n=11)	-	-	At times 1, 2, 3 for released players: 2.64; 3.82; 3.36.	Proportion of deselected players experienced higher levels of psychological distress than retained players at postselection time points; psychological distress was reduced in retained players over time and did not change in released players at postselection time points.
**Brown et al., 2017** ^[25]^	Observational, cross-sectional	Compare mental health between former professional rugby players who were and weren’t forced to retire	Retired profession al rugby union players; Age (38 ± 5); Age at retirement (31 ± 4); Career length (8.9 ± 3.8); Retirement (7.4 ± 4.4); France, Ire land, South Africa	293: 173 (Voluntary retirement), 120 (Forced retirement); 100/0	NS	NS	29% (overall); 26% (retired voluntarily); 32% (forced retirement)		-	-	-	The overall prevalence for anxiety/depression was 29%. The prevalence for forced retirement was 32%; and 26% for voluntary retirement. The prevalence between forced retirement and voluntary retirement was not significantly different.
**Foskett et al., 2017** ^[9]^	Observational, cross-sectional	Prevalence of signs of anxiety/depression and distress	Sample of elite athletes from various individual and team sports competing at a professional, international or national level; Age (24 ± 8.6); UK	143; 57/43 (1 participant did not specify gender)	GHQ method	2	48%		Career dissatisfaction*; severe injuries	GHQ score	-	48% prevalence of anxiety/depression; career dissatisfaction was a significant independent predictor of signs of anxiety/depression.
**Gouttebarge et al., 2015a** ^[31]^	Observational, cross-sectional	Mental and psychosocial health	Current (mean age 27 ± 5) and former footballers (mean age 36 ± 5); duration of career: current (9 ± 5); former (12 ± 5); duration of retirement (5 ± 3); 60% play/played in the highest leagues; Australia, Ireland, The Netherlands, New Zealand, Scotland and USA	253: 149 (current) and 104 (former); 100/0	GHQ method	2	26% (current); 39% (retired)		Severe injuries; surgeries; LE <12*; LE >12; low social support from trainer/ supervisor; low social support from teammates/colleagues*	GHQ score	-	26% and 39% prevalence of anxiety/depression in current and former football players respectively. Recent adverse life events were significantly associated with anxiety/depression in current and former footballers; low social support from teammates was also significantly associated with anxiety/depression in current footballers.
**Gouttebarge et al., 2015b** ^[27]^	Observational, cross-sectional	Prevalence and determinants of symptoms related to mental disorders	Retired professional footballers; mean age 35; duration of career (12 ± 5); duration of retirement (4 ± 4); various countries	219; 100/0	GHQ method	2	35%		Severe injuries; surgeries; LE <6*; LE >6; career dissatisfaction	GHQ score	-	35% prevalence of anxiety/depression. Significant positive association of recent life events with anxiety/depression.
**Gouttebarge et al., 2015c** ^[30]^	Observational, cross-sectional	Symptoms of common mental disorders in professional football	Professional footballers; Finland, France, Norway, Spain, Sweden	540: 121 (Finland), 81 (France), 119 (Norway), 70 (Spain), 149 (Sweden); 100/0	GHQ method	2	25–43%	-	LE <6; LE >6; career dissatisfaction	GHQ score	-	25–43% prevalence of anxiety/depression (Spain-Norway); Significant correlation of life events and career dissatisfaction with anxiety/depression (Finland, Sweden).
**Gouttebarge et al., 2015d** ^[16]^	Observational, cross-sectional	Severe musculoskeletal injuries and symptoms of CMD	Professional footballers (mean age 27; 54% playing in the highest professional leagues); Finland, France, Norway, Spain, Sweden	540; 100/0	GHQ method	2	37%	-	Severe injuries; severe joint injuries; severe muscle injuries; surgeries	GHQ score	-	37% prevalence of anxiety/depression. The number of severe musculoskeletal injuries positively correlated with anxiety/depression. However, no significant associations with severe injuries/surgeries and anxiety/depression.
**Gouttebarge et al., 2015e** ^[26]^	Observational, prospective cohort	Symptoms of common mental disorders and adverse health behaviours	Professional footballers (mean age 27; 55% playing in the highest professional leagues); various	607; 100/0	GHQ method	2	38%	-	Severe injuries; surgeries; LE <6; LE >6; career dissatisfaction*	GHQ score	-	Prevalence of 38% for anxiety/depression. Statistically significant correlations were found for severe injuries and career dissatisfaction with anxiety/depression.
**Gouttebarge et al., 2016a** ^[17]^	Observational, cross-sectional	Prevalence and determinants of symptoms of CMD	Retired professional rugby union players; mean: age 38; career length 9; retirement duration 8; France, Ireland and South Africa	295; 100/0	GHQ method	2	28%	-	LE <6*; LE >6; career dissatisfaction	GHQ score	-	Anxiety/depression prevalence of 28%; a higher number of adverse life events was associated with anxiety/ depression. Significant negative correlation with career dissatisfaction and anxiety/ depression.
**Gouttebarge et al., 2016b** ^[34]^	Observational, prospective cohort	Symptoms of CMD	Professional footballers (mean age 27; mean career duration of 8 years; 55% playing in the highest leagues)	384 at baseline and 262 at follow up; 100/0	GHQ method	3	32%	37%	Adverse life events; conflict with trainer/coach; career dissatisfaction	GHQ score	-	At baseline, there was a 32% prevalence of anxiety/ depression; there was a 12-month incidence of 37% anxiety/depression. Although not statistically significant, there was an association between adverse life events and career dissatisfaction with anxiety/depression.
**Gouttebarge et al., 2016c** ^[29]^	Observational, cross-sectional	Relationship of level of education and employment with symptoms of common mental disorders	Current (mean age 27) and Retired (mean age 35) professional footballers; career duration: current (7.8 ± 4.4); retired (11.6 ± 5.0); Belgium, Chile, Finland, France, Japan, Norway, Paraguay, Peru, Spain, Sweden and Switzerland	607 current and 219 retired; 100/0	GHQ method	2	38% (current); 35% (retired)	-	Level of education; employment status; working hours	GHQ score	-	Anxiety/depression prevalence of 38% and 35% among current and retired football players respectively. Significant negative correlations between employment status and number of hours work with anxiety /depression among retired players.
**Gouttebarge et al., 2016d** ^[32]^	Observational, cross-sectional	Prevalence and risk indicators of symptoms of common mental disorders	Current (mean: age 27; career duration 8) and former (mean: age 50; career duration 11; retirement duration 20) elite athletes from various sports; The Netherlands	485: 203 (current) and 282 (former); 36/64 (current); 49/51 (former)	GHQ method	3	45% (current); 29% (former)	-	Severe injuries; surgeries; recent adverse life events; career dissatisfaction; support	GHQ score	-	45% and 29% prevalence of anxiety/depression among current and former athletes respectively. Current and former athletes with a higher number of severe injuries, higher number of surgeries, higher number of recent adverse life events, higher level of career dissatisfaction will be more likely to report symptoms of anxiety/depression.
**Gouttebarge et al., 2016e** ^[40]^	Observational, prospective cohort	Epidemiology of CMD	Elite Gaelic athletes (hurlers and footballers); mean: age 25; career duration 5; Ireland	204 at baseline and 108 at follow up; 100/0	GHQ method	3	48%	21%	Severe injury*; surgeries; recent life events; career dissatisfaction	GHQ score	-	48% prevalence for anxiety/depression; 6-month incidence of 21% for anxiety/ depression. Significant association with severe injury and the 6-month incidence for anxiety/depression.
**Gouttebarge et al., 2017a** ^[38]^	Observational, prospective cohort	Symptoms of common mental disorders	Elite athletes from various sports; mean age 27 years; mean career duration 8 years; The Netherlands	203 at baseline and 143 at follow up; 36/64	GHQ method	3	45%	57%	Being injured; recent adverse life events; career dissatisfaction	GHQ score	-	At baseline, there was a 45% prevalence of anxiety/depression; 12-month incidence of anxiety/depression was 57%. No statistically significant associations, but career dissatisfaction did increase the likelihood of symptoms of anxiety/ depression by 3.5 times.
**Gouttebarge et al., 2017b** ^[39]^	Observational, prospective cohort	Symptoms of common mental disorders	Current (mean age 26; career duration 8 years) and retired (mean age 35; career duration 11 years) professional ice hockey players	258 (135 current and 123 retired players) at baseline and 158 (81 current and 77 retired players) at follow up; 100/0	GHQ method	3	24% (current); 19% (retired)	17% (current); 8% (retired)	Severe injuries; surgeries; recent adverse life events; career dissatisfaction; support	GHQ score	-	There was a prevalence of 24% and 19% of anxiety/depression for current and retired athletes respectively. The incidence of symptoms of anxiety/ depression was 17% and 8% for current and retired athletes respectively. Although not statistically significant, severe injuries, recent adverse life events and career dissatisfaction increased the likelihood of reporting symptoms of anxiety/depression in current and retired ice hockey players.
**Gouttebarge et al., 2017c** ^[28]^	Observational, cross-sectional	A history of concussions is associated with symptoms of common mental disorders	Former professional athletes (football, ice hockey and rugby union); mean: age 37; career duration 10; years retired 7; Finland, France, Ireland, Norway, South Africa, Spain, Sweden, Switzerland	576; 100/0	GHQ method	3	26% (whole group); 26% (football); 18% (ice hockey); 28% (rugby)	-	Number of concussions	GHQ score	-	Whole group prevalence of 26% anxiety/depression; 26%, 18% and 28% prevalence anxiety/ depression for football, ice hockey and rugby players respectively. There was a significant difference in the number of concussions when groups were divided by presence/absence of symptoms of anxiety/depression (presence having a higher number of concussions). Former athletes reporting 4 or 5 concussions were nearly 1.5 times as likely to report symptoms of anxiety/depression; those reporting 6 or more were two times as likely to report symptoms. This relationship was found across all three sports individually as well.
**Gouttebarge et al., 2018** ^[37]^	Observational, prospective cohort	Symptoms of CMD	Professional rugby union players; mean: age 26; career duration 6; Australia, England, France, Ireland, Italy, New Zealand, Pacific Islands, South Africa and Wales	595 at baseline and 333 at follow up; 100/0	GHQ method	3	32%	28%	Adverse life events; rugby career dissatisfaction	GHQ score	-	At baseline, there was 32% prevalence of anxiety/depression; there was a 28% incidence of anxiety/depression during the 12-month follow up. Although not statistically significant, recent adverse life events or career dissatisfaction were associated with the incidence of anxiety/ depression.
**Hulley et al., 2007** ^[18]^	Observational, cross-sectional	Eating disorders in elite female distance runners	Elite distance runners from the UK and Kenya aged between 15–30 (20.8 ± 3.7)	Athletes (82 UK and 75 Kenyan) and Non-athlete controls (97 UK and 101 Kenyan); 0/100	Likert-scale	-	-	-	-	-	UK athletes and cont rols (20.6 ; 21.4); Kenyan athletes and control (15.9; 17.3)	Kenyan athletes scored lower than UK groups on the GHQ; athletes scored lower than the controls. Kenyan runners were less likely to report symptoms of anxiety/depression than UK runners.
**Ivarsson et al., 2015** ^[41]^	Observational, prospective cohort	Predictive ability of perceived talent devel opment environment (TDE) on well-being	Elite Swedish youth footballers aged 13–16 years old (14.16 ± 1.00)	195; 100/0	Likert-scale	-	-	-	GHQ score	TDEQ (Quality of talent developmental environment)	-	A high quality TDE appears to be associated with higher levels of self-reported wellbeing among youth football players; players experiencing the lowest perceived TDE quality reported the lowest level of well-being over time.
**Kilic et al., 2017** ^[19]^	Observational, cross-sectional	Symptoms of common mental disorders and related stressors	Professional football and handball players (current and retired); Age current/retired football players (25.8 ± 4.9/34.0 ± 4.9); Age current/retired handball players (25.3 ± 4.5/35.0 ± 5.6); Denmark	348 current & 345 retired football players ; 232 current & 230 retired hand ball players; 82/18 (Current foot ballers) ; 79/21 (Retired foot ballers); 51/49 (Current hand ball players); 100/0 (Retired handball players)	GHQ method	2	18% (current footballers); 19% (retired footballers); 26% (current handball players); 16% (retired handball players)	-	Severe injuries*; surgeries*; recent adverse life events*	GHQ score	-	A prevalence of 18% and 19% anxiety/depression was observed for current and retired football players respectively; a prevalence of 26% and 16% anxiety/ depression was observed for current and retired handball players respectively; significant associations between a higher number of recent adverse life events and risk of anxiety/ depression in all athletes; in retired football players there were significant associations with a higher number of severe injuries and surgeries with risk of anxiety/depression.
**Kilic et al., 2018** ^[42]^	Observational, prospective cohort	Severe musculoskeletal time-loss injuries and symptoms of common mental disorders	Professional footballers from national player unions; Age (27 ± 5); duration of career (8 ± 5); Finland, France, Norway, Spain, Sweden	384 at baseline and 262 at follow up; 100/0	GHQ method	3	32%	-	GHQ score; Musculoskeletal time-loss injuries	Musculoskeletal time-loss injuries; GHQ score	-	32% prevalence of anxiety/depression at baseline; not associated with the incidence of severe musculoskeletal time-loss injury. However, musculoskeletal injuries at baseline were associated with the incidence of anxiety/depression in the follow up period even when adjusted for age and adverse life events.
**Niazi et al., 2014** ^[20]^	Observational, cross-sectional	Relationship between emotional intelligence and mental health in collegiate champions	Collegiate athletes of Islamic Azad University; Age (22.35 ± 2.49); history of sports in years (9 ± 2); various sports; Iran	192; NS	NS	NS	-	-	Self-control*	GHQ score	-	Significant positive correlation between emotional intelligence and mental health; self-control explains 76% of the variations in mental health.
**Noblet et al., 2003** ^[21]^	Observational, cross-sectional	Predictors of the strain experienced by professional Australian Footballers (Aussie rules)	Professional Australian football players	255; 100/0	Likert-scale	-	-	-	Post-football uncertainty*; social support from work*; pressure to perform*	GHQ score	-	Post-football uncertainty, social support from work and the constant pressure to perform were significant predictors of psychological health.
**Peretti-Watel et al., 2004** ^[22]^	Observational, cross-sectional	Risky behaviour among elite student athletes	Elite student athletes competing at regional, national or international/Olympic level; age (18.3); France	458; 65/35	Likert-scale	-	-	-	Smoking; Cannabis use	GHQ score	Girls (15.2 ± 6.9); Boys (11.3 ± 5.3)	Girls had a significantly higher GHQ score than boys. GHQ scores were significantly correlated with smoking and cannabis use among elite student athletes.
**Quarrie et al., 2001** ^[43]^	Observational, prospective cohort	Association between potential risk factors and injury risk	Professional rugby union players; age (20.6 ± 3.7); New Zealand	250; 100/0	NS	NS	-	-	GHQ score	Injury	-	No significant association of GHQ score with injury risk.
**Schuring et al., 2017a** ^[23]^	Observational, cross-sectional	Association between osteoarthritis (OA) and symptoms of common mental disorders	Former elite athletes (rugby, football, ice hockey, Gaelic sports and cricket); age (37 ± 6); duration of career (10 ± 5); duration of retirement (6 ± 5); Finland, France, Ireland, Norway, South Africa, Spain, Sweden and Switzerland	With OA (200) and Without OA (402); 100/0	GHQ method	4	31% (with OA); 25% (without OA)	-	OA*	GHQ score	-	OA might be a risk factor for developing symptoms of CMD in former elite athletes. Prevalence of 31% and 25% for retired athletes with OA and those without OA respectively. OA was significantly associated with having more than 2 CMDs. Further, there was a significant association of OA with anxiety/depression in former ice hockey players.
**Schuring et al., 2017b** ^[44]^	Observational, prospective cohort	Mental well-being of current and retired professional cricketers	Current (age: 27 ± 5) and former professional cricketers (age: 36 ± 6); duration of career (current: 6 ± 5), (former: 12 ± 6); South Africa	116: 78 (current) and 38 (former) at baseline; 91/9	GHQ method	2	37% (current); 24% (retired)	15% (current)	Significant injuries; surgeries; adverse life events*; career dissatisfaction*	GHQ score	-	Prevalence of 37% and 15% incidence over 6 months for anxiety/ depression in current cricketers; prevalence of 24% for anxiety/ depression in former cricketers. Career dissatisfaction and adverse life events had positive associations with anxiety/ depression in current cricketers.
**Sekizaki et al., 2017** ^[45]^	Randomised controlled trial	Effectiveness and contribution of internet-based Cognitive Behavioural Therapy (iCBT) to mental healthcare in a school setting	High school athletes competing at a national level in various sports; Japan	80: 40 intervention group and 40 controls; 100/0	GHQ method	NS	-	-	-	-	Pre: Intervention and control (3;3.3); Post: Intervention and control (1.6; 2.2)	School mental healthcare programme using iCBT is suitable for students and useful for coping with stress and reducing depressed mood and anxiety (lower GHQ scores) in young people, especially athletes.
**Stephan, 2003** ^[35]^	Observational, prospective cohort	Repercussions of transition out of elite sport on subjective well-being	Olympic athletes (active and retired); athletes ages ranged from 27 to 35 years (retired: 31 ± 4; active: 29 ± 2); France	32: 16 retired and 16 active; 50/50	Likert-scale	-	-	-	-	-	Time 1, 2, 3: 25.06; 21.18; 19.56	At the time of retirement, retired athletes exhibited significantly decreased subjective well-being (mean GHQ score = 25.06) compared to active athletes (mean GHQ score = 21.18). However, as transitional athletes became used to retirement, their well-being increased over time (mean GHQ score = 19.56).
**Totterdell et al., 2001** ^[24]^	Observational, cross-sectional	Negative mood regulation (NMR) expectancies and sports performance	Professional cricketers from county cricket clubs; ages ranged from 17 to 38 years (25 ± 5); England	46; 100/0	Likert-scale	-	-	-	-	-	10.04 ± 5.21	Mean GHQ score (10.04) was not significantly correlated with performance over a season; it was significantly correlated with NMR, which itself is significantly correlated with performance. Furthermore, NMR did not correlate with performance when well-being was covaried for.
**van Ramele et al., 2017** ^[36]^	Observational, prospective cohort	Incidence of symptoms of common mental disorders	Retired professional footballers; age (35 ± 6); duration of career (12 ± 5); duration of retirement (4 ± 3); Various	212 (baseline) and 194 (follow up); 100/0	GHQ method	3	-	29%	Adverse life events	GHQ score	-	Highest incidence over 12 months was anxiety/depression (29%). However, there was no association between adverse life events and anxiety/depression. 96% of retired football players agreed that mental health can influence players during their career while more than half agreed that mental health affected their performance. More than 80% agreed that there is insufficient medical support for mental health for both current and retired footballers
**Wilson et al., 2015** ^[46]^	Quasi-experimental	Effects of a diet and exercise intervention on body composition, metabolism, bone and mental health	Highly experienced jockeys; UK	10; NS	GHQ method	4	21%	-	-	-	Pre: 10.3 ± 4.3; Post: 8.9 ± 3.8	There was a 21% prevalence of jockeys suffering from symptoms of anxiety/depression. Mean GHQ-12 score was 10.3 prior to the dietary intervention reducing to 8.9 post-intervention. However, this was not statistically significant.

* indicates results are significant (p<0.05);

LE, life event; NS, not specified.
